# Metastatic Spreading of Community Acquired *Staphylococcus aureus* Bacteraemia

**DOI:** 10.1155/2011/234018

**Published:** 2011-07-03

**Authors:** Giovanna Fabio, Maria Carrabba, Luca Mellace, Cinzia Hu, Diego Spagnoli, Maria Domenica Cappellini

**Affiliations:** ^1^Department of Internal Medicine, Università degli Studi di Milano and Fondazione IRCCS “Cà Granda” Ospedale Maggiore, 20122 Milano, Italy; ^2^Department of Neurological Sciences, Università degli Studi di Milano and Fondazione IRCCS “Cà Granda” Ospedale Maggiore, 20122 Milano, Italy

## Abstract

A 29-year-old woman presented to the Fondazione IRCCS “Cà Granda” Ospedale Maggiore, a tertiary care university hospital in Milan (Italy), with skin lesions, fever, myalgia, joint pain and swelling, and a one-week history of low back pain. The diagnosis was *Staphylococcus aureus* (*S. aureus*) bacteraemia spreading to skin, bones, and joints and a lumbosacral epidural abscess L5-S2. Neither initial focus nor predisposing conditions were apparent. The antibiotic therapy was prolonged for six-weeks with the resolution of fever, skin lesions, articular inflammation, and the epidural abscess. Community-acquired *S. aureus* infections can affect patients without traditional healthcare-associated risk factors, and community acquisition is a risk-factor for the development of complications. Raised awareness of *S. aureus* bacteraemia, also in patients without healthcare-associated risk factors, is important in the diagnosis, management, and control of this infection, because failure to recognise patients with serious infection and lack of understanding of empirical antimicrobial selection are associated with a high mortality rate in otherwise healthy people.

## 1. Introduction

Rates of *Staphylococcus aureus *(*S. aureus*) bacteraemia remain high around the world and represent a significant healthcare burden. In the United States, excess costs, length of hospitalisation, and inpatient deaths due to *S. aureus* are estimated at $9.5 billion, 2.7 million days, and 12 000 patients per year, respectively [1]. 

Community-acquired *S. aureus *infections can affect patients with few or without traditional healthcare-associated risk factors, and community acquisition is a risk-factor for the development of complications [2].

In spite of advances in medical care and discovery of potent antistaphylococcal antibiotics, about a third of patients with *S. aureus *bacteraemia die within 30 days [3]. The risk of death is similar for methicillin sensitive and methicillin resistant *S. aureus *infections [4]. The most critical determinants of survival and reduction of complications are an early identification of bloodstream infections, combined with the induction of timely and appropriate antibiotic therapy [2]. Early recognition, prompt initiation of appropriate antibiotics, and rapid microbiological diagnosis are, therefore, key components of effective clinical management. 

Raised awareness of *S. aureus *bacteraemia, also in patients without healthcare-associated risk factors, is important in the diagnosis, management, and control of this infection, because failure to recognise patients with serious infection and lack of understanding of empirical antimicrobial selection are associated with a high mortality rate.

## 2. Case Presentation

A 29-year-old previously healthy woman was admitted to Emergency Department because of sudden appearance and spreading of skin lesions, fever, myalgia, joint swelling, and a painfully decreased range of joint motion. She had one-week history of low back pain for which her physician prescribed a three-day course of intramuscular nonsteroidal anti-inflammatory drug (NSAID). 

**Figure 1:**
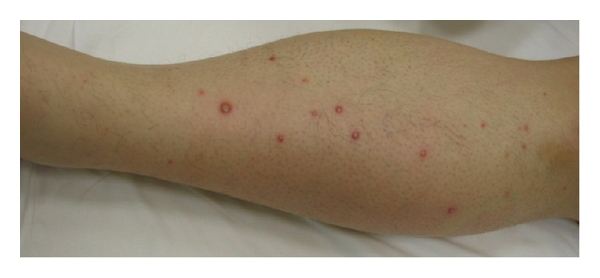
*Skin vesiculobullous lesions *on left leg.

**Figure 2:**
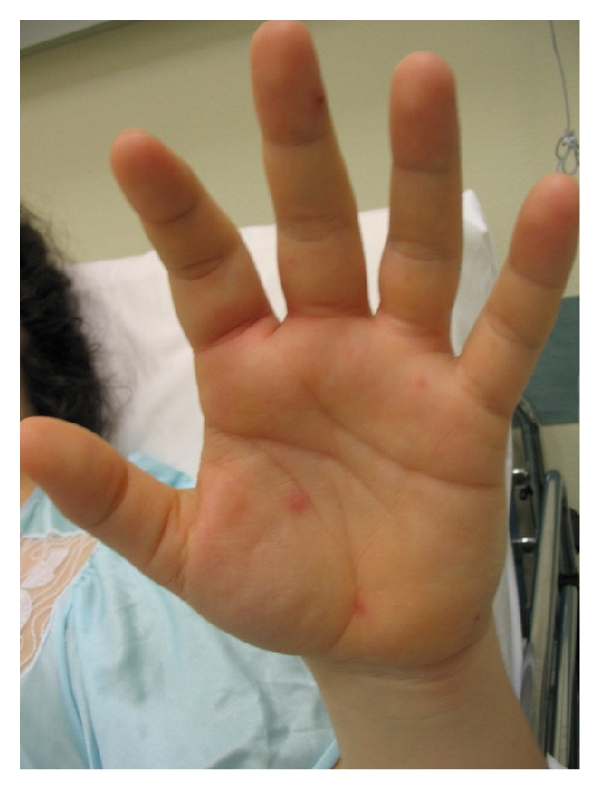
*Skin lesions *on left palm and on third finger.

The patient was overweight (body mass index, BMI = 44), her medical history was unremarkable, she did not have toxic habits and she had no travelled abroad. On admission to our Department blood pressure was 110/80 mmHg, pulse 120 beats per minute, temperature 38.5°C. On physical examination, she presented vesiculobullous lesions (Figure 1) mostly located on her trunk and extremities, including palms and soles (Figure 2). None were seen on her lips or oropharynx.

**Figure 3:**
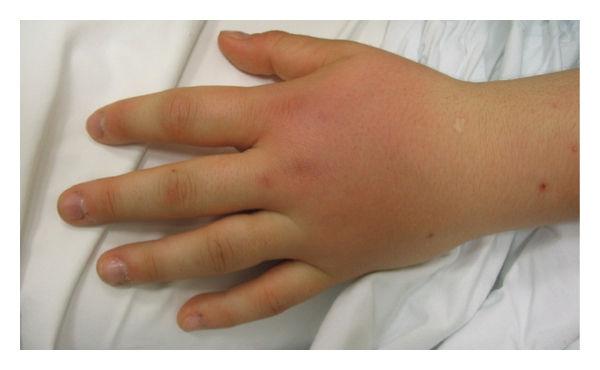
*Metacarpal-phalangeal arthritis* of the left hand.

An asymmetric arthritis of her left elbow, wrist and metacarpophalangeal joints (Figure 3), right ankle, and first metatarsophalangeal joint was present. The sites of the previous intramuscular injections were undetectable. Leukocytosis (white blood cell count 27.5 × 10^9^ cells/L with 91% Neutrophils) elevated level of lactic dehydrogenases, transaminases, alkaline phosphatase, and raised C-reactive protein concentration were found. A chest X-ray revealed no evidence of pulmonary disease, and an electrocardiogram was normal.

Given the clinical picture, strongly suspected for systemic infection, empirical antibiotic treatment was started after microbiological studies, and intravenous ceftriaxone administered at 2 g per day. 

Serologic tests were negative for acute viral infections (hepatitis, epstein-barr, cytomegalovirus, herpes zoster and simplex viruses, and HIV included), rickettsiosis, and borreliosis. 

A methicillin susceptible *S. aureus *was isolated from blood and aspirate of fluid from bullae. 

**Figure 4:**
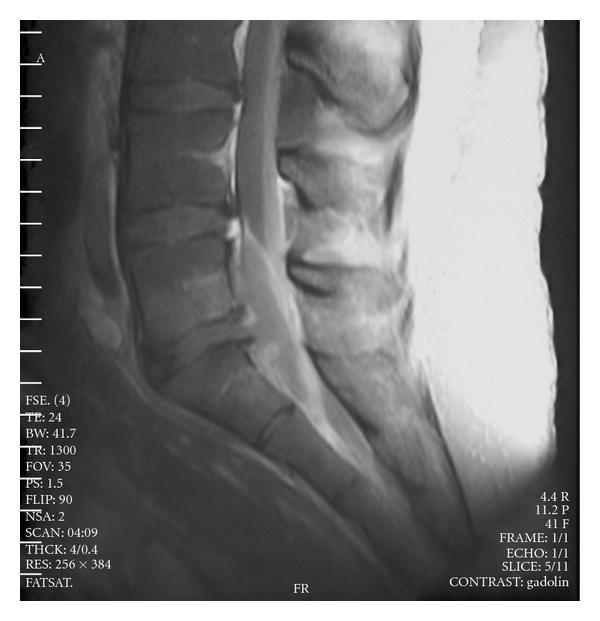
Sagittal T2-weighted, gadolinium-enhanced NMR imaging demonstrates enhancing *epidural collection* ventrally compressing the thecal sac opposite the L5-S2 vertebrae. The L5-S1 disk space is partially obliterated and the bony end plates enhanced, reflecting *discitis and osteomyelitis*.

An epidural soft extra-mass, highly intense to T2 signal nuclear magnetic resonance (NMR) imaging, brightly enhanced by gadolinium injection, was seen to adhere the spinal cord close to L5-S2 associated to a very beginning spondylodiscitis L5-S1 (Figure 4).

A transesophageal echocardiography showed a normal ejection fraction and no evidence of valvular disease or vegetations suggestive of endocarditis.

The patient was diagnosed with *S. aureus *bacteraemia spreading to skin, bones, and joints and a lumbosacral epidural abscess L5-S2. 

Fever disappeared, and skin lesions cleared up in a week, but antibiotic therapy was prolonged for six weeks considering the spread over of the infection and the epidural abscess. No neurological signs manifested, and the articular inflammation gradually subsided. NMR imaging evaluation was performed every two weeks until the resolution of epidural abscess. Plain X-rays of the involved joints after symptoms resolutions did not show joint destructive changes.

## 3. Discussion

*S. aureus *bacteraemia is common and increasing worldwide and is associated with substantial morbidity and mortality. It is often associated with a local focus of infection that has gained access to the bloodstream but in about one-third of patients (notably in community acquired cases), no initial focus is apparent. 

Fever and severe myalgias are usually present in symptomatic cases, and overt septic shock may develop. Otherwise, low-grade bacteraemia leading to seeding of heart valves or other sites may be totally asymptomatic. Metastatic infections usually result from haematogenous seeding of a deep site (e.g., endocarditis, septic arthritis, splenic abscess, spondylodiscitis, and skin/soft tissue), may be clinically obvious or may be occult and totally asymptomatic, and can manifest early in the illness or weeks later.

The presence of *S. aureus *bacteraemia does not, in and of itself, establish the source of infection, because *S. aureus *is also the cause of a number of mimicking conditions such as osteomyelitis, discitis, sepsis, and endocarditis.

The disease of our patient fitted the Center for Disease Control (CDC) definition for community acquisition [5], because MSSA was diagnosed in an outpatient with no history of hospitalization in the past year, or surgery, or other form of hospital-facilities care, no indwelling catheters, or medical devices carrier.

 The bacterial infection of the skin was the reason to seek medical advice and were an effect of the bloodstream infection spreading.

The primary and most important step in the assessment of a patient with *S. aureus *bacteraemia is defining the extent of infection, as this will determine the nature of management. A very active search for all complicating infectious foci should be recommended, since 32% of complicating infectious foci did not have guiding signs and symptoms and 10% are first diagnosed at autopsy. Infection-related mortality is 29% in patients with complicating infectious foci [6].

Factors associated with the development of complicating infectious foci or high mortality are a delay in treatment for more than 48 h after the onset of symptoms, community acquisition, persistently positive blood cultures, persistent fever, congenital heart disease, and the presence of foreign bodies or prosthetic valves and underlying conditions such as immunosuppression and malignancy [6].

The BMI of our patient was 44. Obesity has not been demonstrated as a risk factor for community acquisition of *S. aureus *bacteraemia, while a recent study showed that obesity, together with smoking, constitutes an important risk factor for case fatality in bacteraemic patients [7].

The low back pain complained first by the patient could be the initial manifestation of the epidural abscess. Spinal epidural abscess is a rare but serious cause of back pain. Classical clinical triad consists of back pain, fever, and neurologic deficit, but it is not always present. Clinical picture is often nonspecific, and physicians can attribute back pain to other more frequent infectious (osteomyelitis, meningitis, urinary tract infections) or noninfectious conditions, as was the case of this patient [8]. The clinical signs and symptoms are subtle, vague, and insidious. High level of awareness is required by physicians in order to making the correct diagnosis in the early stage of disease to avoid the severely debilitating complications from delayed or inadequate treatment.

Bacteria gain access to the epidural space through contiguous spread (about one third of cases) or haematogenous dissemination (about half of cases); in the remaining cases, the source of infection is not identified. Predisposing factors include spinal surgery, intravenous drug use, immunosuppression, and epidural catheterism; however, in up to one third cases, no risk factors are present. Because most predisposing conditions allow for invasion by skin flora, *S. aureus *causes about two thirds of cases of spinal epidural abscess [9]. Likewise, infection that originates in the spinal epidural space can extend locally or through the bloodstream to other sites. Discitis and osteomyelitis coexist with spinal epidural abscess in up to 80% of patients [10]. The incidence of acute haematogenous nontuberculous vertebral osteomyelitis was estimated to be five cases per million patients per year [11].

NMR imaging with intravenous administration of gadolinium and myelography is the imaging method of choice with more than 90% sensitivity in diagnosing spinal epidural abscess and is especially useful in the early stages of infection when other imaging modalities are still normal (radiography). It delineates both the longitudinal and paraspinal extension of the abscess and may help differentiate infection from cancer on the basis of the appearance and the signal intensity of the image [12]. A plain roentgenograph or CT-scan of the spine may reveal narrowing of the disk and bone lysis to indicate the presence of discitis and osteomyelitis. 

Patients with *S. aureus *bacteraemia can develop secondary septic arthritis, particularly if an underlying joint disease such as rheumatoid arthritis is present. The joints affected, in decreasing order of frequency, are the knee, hip, elbow, shoulder, and interphalangeal joint. The arthrocentesis to evaluate for septic arthritis was not performed in the patient we described, and then, the infectious etiology of arthritis was not demonstrated. Thus, the patient presented polyarticular arthritis and no underlying joint disease such as rheumatoid arthritis was present, suggesting a different etiology. Induction of arthritis during *S. aureus *infection might be also triggered by the concerted action of both superantigens activating T lymphocytes and exposure to peptidoglycans/capsular polysaccharides and free bacterial DNA (originating from disrupted bacteria) triggering macrophages to release proinflammatory cytokines [13].

Patients presenting with *S. aureus *bacteraemia complicated by metastatic infection will require prompt intravenous administration of appropriate antibiotics that should be prolonged for 4 to 6 weeks. Surgery is mandatory if neurological dysfunction develops in patients with spinal epidural abscess. Otherwise, antibiotic therapy alone and close followup is a valuable choice if the microbial cause is identified and the patients' clinical condition is closely monitored [14]. Neurologic function, signs of sepsis, and imaging findings should be closely monitored after treatment begins. A neurologic deterioration between admission and accurate diagnosis may lead to a poorer outcome [15].

The inability to rapidly identify and characterise infecting organisms means that initial antibiotic therapy is often empirical. This may result in inappropriate treatment, which is associated with extended overall duration of hospitalisation, increased risk of patient mortality and increased overall cost of treatment particularly for patients infected with methicillin-resistant *S. aureus*. The traditional approach to treatment of staphylococcal infections is to prescribe first generation cephalosporins (cefazolin, cephalothin, and cephalexin), clindamycin, lincomycin, and erythromycin for outpatients in less serious methicillin-susceptible *S. aureus *infections such as skin and soft tissue infections. Penicillinase-resistant penicillins (flucloxacillin and dicloxacillin) remain the antibiotics of choice for the management of serious methicillin-susceptible *S. aureus *infections for patients requiring hospitalization and vancomycin for patients with *β*-lactam allergies or methicillin-resistant strains of *S. aureus* [16, 17]. However, with the emergence and increasing prevalence of community-associated methicillin-resistant strains [18], some of these choices may need reconsideration. From a clinical standpoint, given the morbidity and mortality associated with delayed treatment of MRSA infection, it would be prudent to include methicillin-resistant *S. aureus *coverage in empirical antibiotic regimens in settings where a significant proportion of patients hospitalized for community-acquired *S. aureus *infection have methicillin-resistant strains. 

A recent review focusing on the evidence behind the key clinical decisions in the management of *S. aureus *bacteraemia highlights just two key principles; that is, all infective foci must be identified and removed as soon as possible, and long-term antimicrobial therapy is required for those with persistent bacteraemia or a deep, irremovable focus. The best drugs, dose, mode of delivery, and duration of therapy remained uncertain, a situation compounded by emerging *S. aureus* strains that are resistant to old and new antibiotics [19].

New management strategies are required including the use of techniques that allow the early identification of complications arising from *S. aureus* bacteraemia, rapid pathogen identification to enable the administration of appropriate antibiotic therapy, and the identification of new drugs that enable effective empirical treatment against both susceptible and resistant *S. aureus*.

Finally, the clinician is again reminded that *S. aureus* can cause severe life-threatening infections in otherwise healthy people.
